# PD-L1, Mismatch Repair Protein, and NTRK Immunohistochemical Expression in Cervical Small Cell Neuroendocrine Carcinoma

**DOI:** 10.3389/fonc.2021.752453

**Published:** 2021-10-21

**Authors:** Longyun Chen, Fan Yang, Ting Feng, Shafei Wu, Kaimi Li, Junyi Pang, Xiaohua Shi, Zhiyong Liang

**Affiliations:** Department of Pathology, State Key Laboratory of Complex Severe and Rare Disease, Molecular Pathology Research Center, Peking Union Medical College Hospital, Chinese Academy of Medical Sciences & Peking Union Medical College, Beijing, China

**Keywords:** Cervix, small cell neuroendocrine carcinoma, targeted therapy, immune checkpoint inhibitor, mismatch repair system, NTRK fusion, PD-L1

## Abstract

**Background:**

Cervical small cell neuroendocrine carcinoma (SCNC) is a rare and aggressive disease that lacks a standard treatment strategy or effective methods of targeted therapy. PD-L1 inhibitors for DNA mismatch repair system-deficient (dMMR) tumors and neurotrophin receptor tyrosine kinase (NTRK) inhibitors offer potential pan-cancer treatments.

**Methods:**

Immunohistochemistry was employed as the main detection method, and any NTRK positive cases, identified by immunohistochemistry, were further submitted for evaluation by fluorescence *in situ* hybridization (FISH) and real-time polymerase chain reaction (RT-PCR) methods.

**Results:**

Forty-six patients were enrolled. Positive PD-L1 expression was seen in 22 of the 43 patients (51.16%) with an average combined positive score of 6.82. PD-L1-positive patients were more likely to have a higher proliferation rate in the tumor, and they experienced less recurrence and death (p = 0.048 and 0.033, respectively) compared with the patients with negative PD-L1 expression. However, in the multivariate analysis, none of the clinical parameters was associated with the expression of PD-L1. There was no association between PD-L1 expression and disease recurrence or overall survival in the Kaplan-Meier analysis. All cases were found to be MMR-stable and lacked NTRK gene fusion. However, pan-Trk expressed in 14 (32.56%) of the 43 tested cases, but FISH and RT-PCR failed to confirm any positive fusion signals in IHC-positive cases.

**Conclusions:**

PD-L1 may be an effective therapeutic target for cervical SCNC. Cervical SCNC is a MMR-stable tumor and lacks NTRK gene fusion. IHC isn’t a reliable method in the detection of NTRK gene fusion in cervical SCNC.

## Introduction

Small cell neuroendocrine carcinoma (SCNC) of the uterine cervix is a rare disease that accounts for less than 1% of cases of cervical cancer (1). It is characterized by easily identified lymphovascular thrombi, early recurrence, and distant metastasis (2). The prognosis is poorer than that of common cervical carcinoma, with an overall survival time of 40 months and a 5 year survival rate of 34% (3). Because of the rarity of this tumor, there are no unique standard treatments, and methods are mainly borrowed from the therapy of the common cervical cancers or small cell neuroendocrine carcinoma of the lung. Radical hysterectomy may be performed, followed by adjuvant chemotherapy in early stage disease in cervical SCNC, while various strategies are applied for locally advanced and metastatic disease, including concurrent chemoradiation, neoadjuvant chemotherapy followed by surgery, or chemotherapy alone (4). Therefore, it would be valuable to find more effective therapeutic methods, especially targeted therapy for SCNC.

Currently, targeted therapy is evolving from a tumor-specific approach towards a more histology-agnostic pattern. PD-L1 inhibitors for DNA mismatch repair system-deficient (dMMR) tumors and neurotrophin receptor tyrosine kinase (NTRK) inhibitors for NTRK fusion positive solid tumors are the most recent representative models for pan-cancer treatment. The PD1/PD-L1 interaction can act as an immune checkpoint by mediating T cell function. Overexpression of PD-L1 in tumor cells or tumor-infiltrated lymphocytes (TILs) can assist the tumor in escaping the immune system. PD-L1 overexpression is also related to the efficacy of the target therapy drugs. Multiple anti-PD1/PD-L1 drugs have been approved for use in solid tumors, including cervical squamous cell carcinoma and small cell lung cancer. PD-L1 expression in tumor cells and/or TILs has been approved as a companion diagnostic marker across different types of tumor, including cervical carcinoma.

The DNA mismatch repair (MMR) system can correct errors that occur during DNA replication, and its deficiency causes the accumulation of mutations in the coding and noncoding microsatellites, a phenotype known as microsatellite instability (MSI). The high tumor burden caused by dMMR can induce more neo antigen expression and attract more TILs, increase the expression of PD-L1, and inhibit the immune response. In recent years, owing to the efficacy of immune checkpoint inhibitors in the treatment of advanced solid tumors with dMMR, in clinical trials, PD-1 inhibitors have been approved for treatment of advanced/recurrent microsatellite instability-high (MSI-H) solid tumors, regardless of the primary tumor site. Hence, MMR status is another biomarker for the selection of anti-PD-L1 drugs. Immunohistochemical test for the expression of four MMR proteins (MSH2, MSH6, MLH1, and PSM2) is a reliable and convenient surrogate for MSI status.

NTRK plays an important role in the physiology of the development and function of the nervous system. Abnormal NTRK fusion can cause ligand-independent activation and proliferation of tumor cells. Patients who are gene-positive for NTRK fusion show an excellent and durable clinical response to targeted receptor tyrosine kinase (RTK) inhibitor therapies, irrespective of the histology type. There are several methods for the detection of NTRK gene fusion, including immunohistochemical (IHC), real-time polymerase chain reaction (RT-PCR), fluorescence *in situ* hybridization (FISH), and next-generation sequencing (NGS). However, to date, there are no approved companion methods for the detection of NTRK gene fusion. The IHC method was reported to have achieved a high concordance with other methods, and it can be used for prescreening in common cancers which have a low frequency of NTRK gene fusion.

In this study, we used immunohistochemistry to evaluate expression of PD-L1, MMR, and NTRK fusion, and explore their correlation with clinicopathological characteristics in cervical SCNC, in order to find more therapeutic targets for this rare type of cervical cancer.

## Materials and Methods

### Patient Information

Forty-six patients were enrolled in our study, which underwent surgery or biopsy between September 2010 and April 2020 at Peking Union Medical College Hospital (Beijing, China). The final diagnosis was made according to the morphology and immunohistochemical characteristics of the tumor. SCNC is composed of a densely monotonous cellular population of round, ovoid, or spindle small cells. The cytologic features include hyperchomatic nuclei with nuclear molding, inconspicuous nucleoli, and scant cytoplasm. Mitotic rates usually exceed 10 per 10 high power field. The diagnosis of SCNC doesn’t require the expression of neuroendocrine markers in the presence of classic morphologic features. Clinical information was retrieved from the digital clinical database, which included clinical symptoms, surgical or biopsy time, surgical type, treatment methods, recurrence, death date, and last follow-up time. Pathological profiles were evaluated by reviewing the hematoxylin/eosin (H&E) slides, and included tumor size, necrosis, mitosis, invasion depth, lymph node metastasis, and lymphovascular invasion.

This study was approved by the Institutional Review Board of Peking Union Medical College Hospital.

### Tissue Microarray Construction

Tissue microarrays (TMAs) were constructed using a manual tissue arrayer (MTA-1; Beecher Instruments, Sun Prairie, WI). Briefly, the representative areas were circled according to the H&E morphology, and 2 cores measuring 2mm in diameter from the donor block were transferred to a blank recipient block.

### IHC

Immunohistochemistry was performed on unstained sections, of 4 μm thickness. The antibodies used in our study included CgA, Syn, ER, PR, Ki-67, P16, PD-L1, MSH2, MSH6, MLH1, PMS2, and panTrk. Immunostaining was performed on a Benchmark ULTRA autostainer or Dako Autostainer Link 48. Cytoplasmic staining in the tumor cells was considered positive for CgA, Syn, and P16. Nuclear staining in the tumor cells was considered positive for ER, PR, Ki-67, MSH2, MSH6, MLH1, and PMS2.

Pan-Trk IHC (EPR 17341, Roche) is considered positive if more than 1% of the tumor cells have staining above the background level. The intensity of immunoreactivity was recorded (weak, moderate, or strong). The pattern of pan-Trk staining (cytoplasmic, nuclear, and/or membranous) was also noted.

PD-L1 (Dako 22C3) expression is determined by the combined positive score (CPS), according to the manual. CPS is calculated by dividing the number of PD-L1 staining cells (tumor cells, lymphocytes, macrophages) by the total number of viable tumor cells, and multiplying the fraction by 100. Any intensity of convincing partial or complete linear membrane staining in the tumor cells was deemed positive, while cytoplasmic staining of the tumor cells was excluded. Any intensity of convincing membrane and/or cytoplasmic staining of the lymphocytes and macrophages within the tumor nests and/or adjacent supporting stroma was included.

### FISH

FISH was performed using the Thermo-Brite Elite automated FISH slide prep system (Leica, Richmond, CA, USA). Three FISH Break Apart Probes, NTRK1, NTRK2, and NTRK3 (ZytoVision GmbH, Bremerhaven, Germany), were used in our study. One hundred tumor nuclei were observed per case, and positive cases were defined as > 15% having split orange and green signals with separation widths of two or more signal diameters, according to the manufacturer’s protocol.

### RT-PCR

The NTRK Gene Fusions Detection Kit (AmoyDx), which can qualitatively detect 109 fusions in NTRK1, NTRK2, and NTRK3, was used according to the manufacturer’s protocol. Three major steps were carried out in sequence, which included RNA extraction, reverse PCR, and DNA amplification. The eight NTRK PCR mix tubes contained fusion detection and internal control systems. The fusion detection system contained primers and FAM-labeled probes specific for NTRK1/2/3 gene fusions. The internal control system contained primers and a VIC-labeled probe for detection of reference genes to reveal the RNA quality and presence of PCR inhibitors that may lead to false negative results. Reverse transcription and amplification PCR were run on an ABI 7500 PCR machine. For the NTRK PCR mix, FAM Ct values ≤ 25 are considered positive. Detailed information of the NTRK fusion types examined by the RT-PCR kit is summarized in the **Supplementary Table**.

### Statistics

The Statistical Package for the Social Sciences (SPSS) Version 20.0.0. (SPSS Inc., Chicago, IL) was used to analyze the data. Chi-square or Fisher’s exact tests were employed to analyze the categorical variables as appropriate. Multivariate analysis was carried out using bivariate regression analysis. Relapse-free survival (RFS) and overall survival (OS) values were calculated using the Kaplan–Meier method. All p values were reported as two-sided, with p values < 0.05 being considered statistically significant.

## Results

### Clinical Characteristics of Cervical SCNC

In total, 46 patients were included in this study, of which eleven were menopausal. Of the 41 patients with known clinical history, 35 presented with vaginal bleeding or abnormal discharge. Five patients were diagnosed upon biopsy, after positive human papilloma virus (HPV) infection on routine physical examination. One patient was diagnosed during a regular gestational checkup. The median age at diagnosis was 42.5 years (range: 24-62 years). Seven patients only underwent biopsy of the tumor, one patient underwent simple uterine hysterectomy and salpingectomy, four patients underwent a uterine hysterectomy and salpingectomy, plus lymphadenectomy. The remaining 34 patients underwent a total pelvic hysterectomy and bilateral salpingo-oophorectomy, plus lymphadenectomy. Positive lymph node metastasis was observed in 14 cases. Stages I, II, III, and IV were recorded in 20 (50.0%), 4 (10.0%), 15 (37.5%), and 1 (2.5%) cases, respectively.

Ten of the 44 patients received chemotherapy before surgery. After surgery, 40 of the 41 patients were treated with chemotherapy, of which 21 were also exposed to radiation therapy.

Follow-up data were available for 42 patients. Overall, 16 (38.10%) patients had a recurrence of disease at an average of 16.81 months (range: 3 – 55.1 months), 11 (26.19%) patients died of the disease at an average of 24.72 months (range: 6.1 – 62.3 months), and 26 (61.90%) patients were disease free.

### Pathological Characteristics of Cervical SCNC

The average tumor size was 2.76cm (range: 0.4 – 7.2cm). Thirty-five cases were pure SCNC, while 11 cases were combined SCNC, including 4 cases combined with squamous cell carcinoma, 1 case of SCNC with squamous cell carcinoma in situ, 3 cases comprised SCNC and adenocarcinoma, and 2 cases were SCNC with adenocarcinoma in situ. One case was composed of SCNC with malignant mixed mullerian tumor. Lymphovascular invasion and tumor necrosis were observed in 32 and 17 cases, respectively. The average mitotic index was 26 per 10 high-power fields (HPF). The involvement of the cervical myometrium was subdivided into less than half of the full thickness (10 cases), and more than half of the cervical thickness (29 cases).

Neuroendocrine markers were positive in all cases, with CgA present in 36 cases and Syn in 45 cases. ER was negative in all cases and PR was positive in only 1 case. Strong and diffuse p16 immunostaining was observed in 45 cases. The average Ki-67 index was 80.43% (range: 5 – 95%).

### PD-L1, MMR, and NTRK Expression in Cervical SCNC

**Figure 1:**
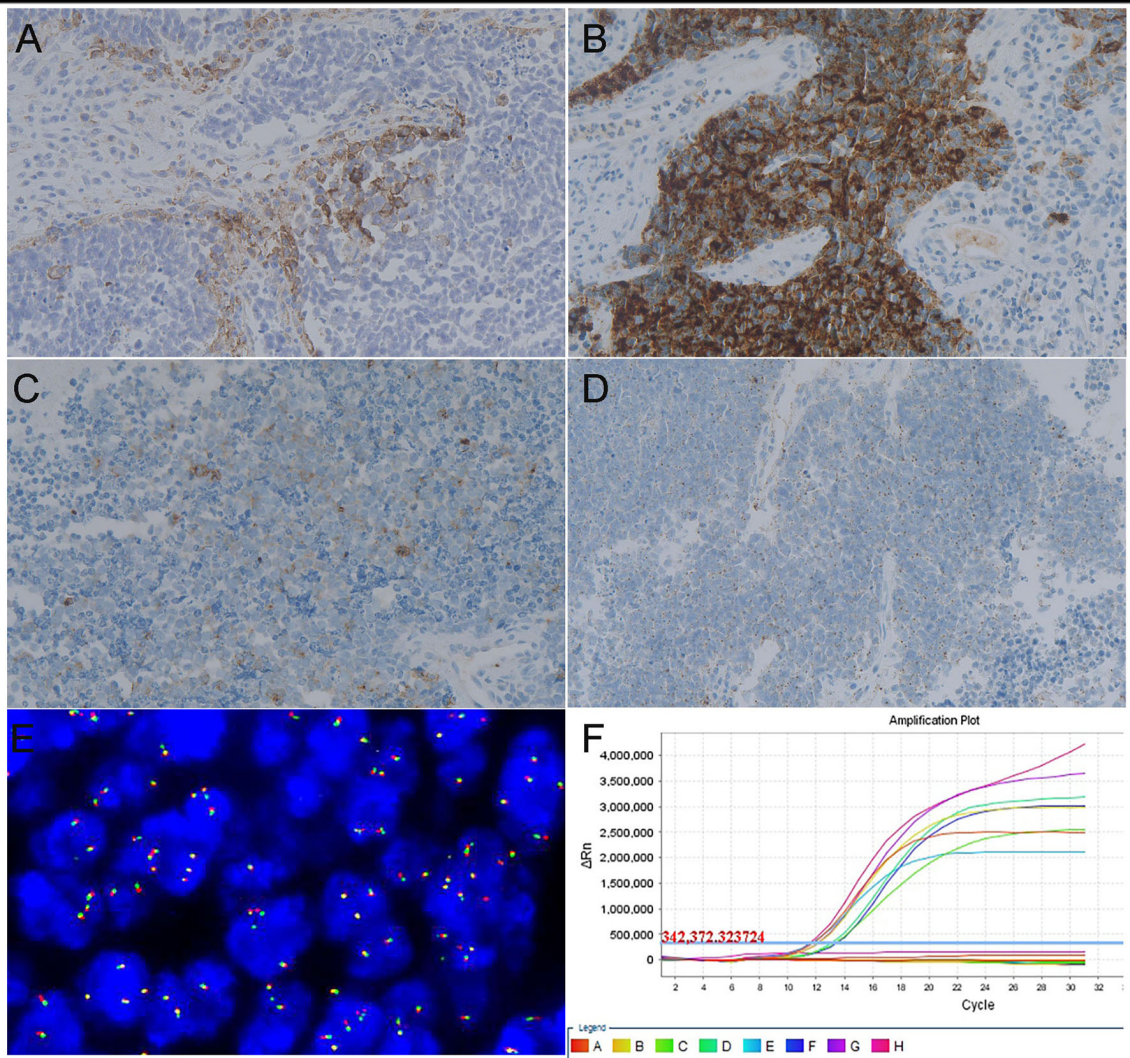
Representative images of targeted therapy markers in cervical SCNC. **(A)** Positive result of PD-L1 examined by immunohistochemistry method (x200). **(B)** Strong intensity result of NTRK fusion gene examined by IHC (x200). **(C)** Moderate intensity result of NTRK fusion gene examined by IHC (x200). **(D)** Weak intensity result of NTRK fusion gene examined by IHC (x200). **(E)** Negative signal of NTRK 1 fusion gene examined by FISH method. **(F)** Negative result of NTRK fusion gene examined *via* RT-PCR method.

Positive PD-L1 expression was seen in 22 (51.16%) of the 43 cases of SCNC. The average CPS score was 6.82 in the positive cases. Positive PD-L1 staining was present only in TILs with an average score of 6.67, while PD-L1 staining appeared in both the tumor cells and immune cells in one case with a score of 10 (**Figure 1A**).

The DNA mismatch repair proteins MLH1, PMS2, MSH2, and MSH6 showed intact nuclear immunoreactivity in all the tested cases, indicating that the MMR status was stable in all cervical SCNC patients in our cohort.

NTRK gene expression was identified in 14 of the 43 tested cases by IHC. There were 5 cases showing strong and diffuse cytoplasmic staining (**Figure 1B**), 5 cases exhibiting moderate cytoplasmic staining (**Figure 1C**), and the remaining 4 cases showed weak cytoplasmic staining in the tumor cells (**Figure 1D**). FISH examination, which was carried out in the IHC-positive cases, revealed that none of them had a positive result of split orange and green signal in the tumor cells (**Figure 1E**). RT-PCR also failed to identify NTRK fusion in any IHC-positive cases (**Figure 1F**). Thus, the final NTRK fusion rate was zero in cervical SCNC, in our cohort.

### Correlation of Clinicopathological Characteristics With PD-L1 Expression in Cervical SCNC

When stratified by PD-L1 expression, PD-L1 positive patients were prone to have an elevated Ki-67 proliferation index (p = 0.034), and the PD-L1 positive patients experienced less recurrence and death (p = 0.048 and 0.033, respectively) compared with the patients with negative PD-L1 expression. No significant correlations were found between PD-L1 expression and patient age, tumor size, clinical stage, lymph node metastasis, tumor necrosis, lymphovascular invasion, or tumor invasion depth. The relationship between PD-L1 IHC expression and clinicopathological characteristics of cervical SCNC patients was summarized in **Table 1**. In the multivariate analysis, none of the clinical parameters were associated with PD-L1 expression (**Table 2**).

**Table 1:** Relationship between PD-L1 IHC expression and clinicopathological characteristics of cervical SCNC patients.

Characterisitcs	No. of patients (Percentage)	PD-L1 IHC		P value
		positive	negative	
***Age***				0.457
>=50	9(20.9%)	6	3
<50	34(79.1%)	16	18
***Mixed or not***				0.132
Yes	9(20.9%)	7	2
No	34(79.1%)	15	19
***Stage***				1.000
Early (I-IIA)	20(54.1%)	11	9
Advanced (IIB-IV)	17(45.9%)	9	8
***Node metastasis***				1.000
Yes	14(40.0%)	8	6
No	21(60.0%)	12	9
***Tumor size (cm)***				0.257
<4	30(78.9%)	15	15
>=4	8(21.1%)	6	2
***LVI***				0.053
Yes	31(77.5%)	20	11
No	9 (22.5%)	2	7
***Necrosis***				0.537
Yes	17(39.5%)	10	7
No	26(60.5%)	12	14
***Mitosis***				0.069
=<20	22(51.2%)	8	14
>20	21(48.8%)	14	7
***Invasion Depth***				1.000
<1/2 depth	7(19.4%)	4	3
≥1/2 depth	29(80.6)	16	13
***Recurrence***				**0.048**
Yes	16(41.0%)	6	10
No	23(59.0%)	16	7
***Death***				**0.033**
Yes	11(28.2%)	3	8
No	28(71.8%)	19	9
***Ki-67 index (%)***				**0.034**
<=75	10(23.3%)	2	8
>75	33(76.7%)	20	13

PD-L1, programmed death ligand 1; IHC, immunohistochemistry; SCNC, small cell neuroendocrine carcinoma; LVI, lymphovascular invasion. Bold values indicate significant statistic difference between the bi-variate factors.

**Table 2:** Multivariate analysis of the correlation between PD-L1 expression and clinicopathological features of cervical SCNC.

Risk factors	p Value	Exp (B)	95.0% CI for Exp(B)	
			Lower	upper
**LVI**	0.132	4.731	0.627	35.699
**Recurrence**	0.867	1.228	0.111	13.542
**Death**	0.259	4.302	0.341	54.278
**Ki-67 index (%)**	0.211	0.273	0.036	2.090
**PD-L1**	0.353	0.270	0.017	4.284

PD-L1, programmed death ligand 1; SCNC, small cell neuroendocrine carcinoma; LVI, lymphovascular invasion.

**Figure 2:**
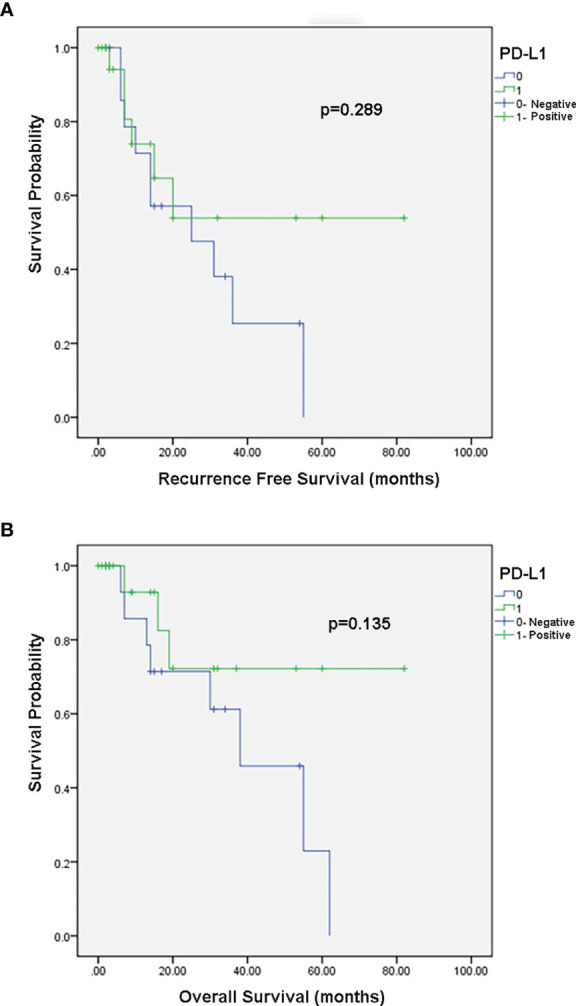
Kaplan–Meier survival curves. **(A)** Association between PD-L1 expression and recurrence free survival. **(B)** Association between PD-L1 expression and overall survival.

There was no association between PD-L1 expression and disease recurrence or overall survival in the Kaplan-Meier analysis (p = 0.289 **Figure 2A** and p=0.135 **Figure 2B**, respectively).

## Discussion

In this study, we found that nearly half of the cases of cervical SCNC showed the positive PD-L1 expression in tumor cells or TILs. PD-L1 positive patients were more likely to have a higher proliferation rate in the tumor, and to have less recurrence and death incidence compared with the negative group. All cervical SCNCs were MMR-stable and lacked NTRK fusion. PD-L1 may be a reliable and effective therapeutic target in cervical SCNC.

Patients with dMMR are good candidates for anti-PD-L1 target therapy. All the patients in our cohort had a mismatch repair stable status examined by IHC. There are different methods for the detection of MSI, while IHC is the most convenient and labor-saving method. Previous results indicated that IHC could achieve a highly concordant result with PCR or NGS (5, 6). Cervical SCNC is a rare type of cervical carcinoma; there are only a few reports focusing on the MMR of cervical SCNC. One study found that 3 out of 9 cases were dMMR in cervical SCNC (7), while the other indicated an MMR stable status in all 28 cases of cervical SCNC (8). Our results are in line with the latter report and ours is also the largest number of cervical SCNC cases investigated, and therefore improves the reliability of MMR data for such rare tumors.

Although immune checkpoint blockade therapies in patients with MSI/dMMR tumors are not practical in cervical SCNC, since all the patients were MMR-stable in our study, PD-L1 expression is another good marker for the selection of immune checkpoint drugs. According to the result of the clinical study KEYNOTE-158, in which pembrolizumab achieved promising clinical efficacy in patients with recurrent or metastatic cervical cancer, the drug has been approved for use in cervical cancer with PD-L1 expression (CPS ≥ 1) by the FDA (9). Knowing that PD-L1 expression is a prerequisite for the selection of a suitable population before applying targeted therapy drugs, we used the same antibody and evaluation criteria as the clinical trial to explore the PD-L1 expression rate in cervical SCNC. PD-L1 expression rate was 51.16% in all the patients and 52.9% in those at advanced clinical stages in our cohort, suggesting that PD-L1 expression is not a rare event and immune checkpoint inhibitors may provide an alternative method of targeted therapy in such a rare but aggressive disease. Several case reports have shown that immune-targeted therapy is effective in cervical SCNC (10, 11). The high positive occurrence of PD-L1 reported in cervical SCNC is valuable, since it can offer these patients another alterative targeted therapy option.

Our study results further illustrate the correlation between PD-L1 expression and the clinicopathological characteristics of cervical SCNC. Although PD-L1 expression was not associated with RFS or OS in our cohort, we could see that the PD-L1 positive patients suffered lower recurrence and death rate in our study. The role of PD-L1 in predicting RFS and OS in cervical tumors is controversial; in cervical carcinoma, it has no impact or acts as an indicator of poor prognosis (12–16). The differing roles of PD-L1 in cervical tumors may originate from factors such as different PD-L1 antibodies and evaluation criteria, different subtypes of cervical carcinoma, and diverse ethnicities.

The NTRK fusion gene, which is another clinically validated, pan-cancer, targeted therapy marker, was also examined in our study. Three different methods (IHC, FSIH, and RT-PCR) were used and the final NTRK fusion rate turned out to be zero in our cohort. NTRK gene fusion is a rare event in common cancers, such as lung cancer, pancreatic cancer, and neuroendocrine tumors (17, 18). NTRK fusion in cervical or uterine sarcoma has been defined as a subtype sarcoma with features of fibrosarcoma (19, 20). To the best of our knowledge, there is only one reported case of NTRK fusion-positive cervical carcinoma (21), and there is no previous study investigating NTRK fusion status in cervical SCNC. During the evaluation process, we found an interesting phenomenon: pan-Trk can show a weak to strong immunostaining pattern in approximately of 32.56% the SCNC specimens, although FISH or RT-PCR methods fail to identify any positive fusion signals in the tumor cells. This questions the use of IHC in assessing NTRK fusion in SCNC. Several studies have investigated the sensitivity and specificity of the pan-Trk IHC method versus the FISH or NGS method, and the results showed that the positive and negative predictive values are high between the various methods, in infant fibrosarcoma, lipofibromatosis-like neural tumor, colorectal cancer, and lung adenocarcinoma. Another group also found that pan-Trk IHC cannot be used as an initial screening method in neuroendocrine tumors because approximately 50% of lesions expressed TRK proteins, in the absence of NTRK gene fusions (22). SCNC originates from the embryonic neuroectoderm and displays an immunohistochemical profile of endocrine and epithelial phenotypes. NSE, which is a highly specific marker for neurons and peripheral neuroendocrine cells, can be expressed in the majority of neuroendocrine tumors. These findings may contribute to the high false positive rate of pan-Trk IHC in neuroendocrine tumors. Pan-Trk IHC is not a reliable tool for the evaluation of NTRK gene fusion in cervical SCNC.

Our study had several limitations. First, since this was a retrospective study, the clinical effect of PD-L1 immune therapy drugs could not be evaluated in cervical SCNC patients. Second, the total sample size was relatively small, owing to the rarity of this tumor. Third, tumor mutation burden, which is reported to be another promising and effective marker to predict immune therapy drugs, was not analyzed in our study. Forth, the use of TMA had limitation in the detection of PD-L1 expression because the heterogeneous of PD-L1 expression in tumor samples.

In conclusion, PD-L1 may be a reliable and effective therapeutic target for cervical SCNC. PD-L1 expression was found in about half of the cervical SCNC cases, indicating that immune checkpoint inhibitors is a promising targeted therapy option for this rare type of tumor. Cervical SCNC is a MMR-stable tumor and lacks NTRK gene fusion. The pan-Trk IHC method showed a high false positive rate of NTRK gene fusion, which questions the use of this method in the detection of NTRK fusion in small cell neuroendocrine tumors.

## Data Availability Statement

The original contributions presented in the study are included in the article/**Supplementary Material**. Further inquiries can be directed to the corresponding authors.

## Ethics Statement

The studies involving human participants were reviewed and approved by Institutional Review Board of Peking Union Medical College Hospital. The patients/participants provided their written informed consent to participate in this study.

## Author Contributions

XS designed and supervised the study. FY collected clinical information of the patients. LC prepared patient samples. TF, FY, and SW analyzed the data. ZL and XS wrote the manuscript. All the authors participated in the interpretation of the results and approved the final version of the manuscript.

## Conflict of Interest

The authors declare that the research was conducted in the absence of any commercial or financial relationships that could be construed as a potential conflict of interest.

## Publisher’s Note

All claims expressed in this article are solely those of the authors and do not necessarily represent those of their affiliated organizations, or those of the publisher, the editors and the reviewers. Any product that may be evaluated in this article, or claim that may be made by its manufacturer, is not guaranteed or endorsed by the publisher.
