# Supplementary material for: PD-L1, Mismatch Repair Protein, and NTRK Immunohistochemical Expression in Cervical Small Cell Neuroendocrine Carcinoma
Source: Front Oncol. 2021 Oct 21;11:752453. doi: 10.3389/fonc.2021.752453 (PMC8566736; doi:10.3389/fonc.2021.752453)
Supplement: Supplementary file 1 [file Table_1.docx]

**SUPPLEMENTARY Table|** Detailed information of the NTRK fusion types examined by the RT-PCR kit.

| **Tube** | **Detected Target** | **Fusion Type** | **Tube** | **Detected Target** | **Fusion Type** |
| --- | --- | --- | --- | --- | --- |
|  | **NTRK1 Fusion** | TP53 exon8;ins6 NTRK1 exon8 | ③ | **NTRK1 Fusion** | TPR exon6;NTRK1 exon12 |
|  |  | TP53 exon9;ins6 NTRK1 exon8 |  |  | GRIPAP1 exon22;NTRK1 exon12 |
|  |  | TP53 exon10;ins6 NTRK1 exon8 |  |  | SCYL3 exon11;NTRK1 exon12 |
|  |  | TP53 exon11;ins6 NTRK1 exon8 |  |  | MEF2D exon9;NTRK1 exon12 |
|  |  | CTRC exon2;NTRK1 exon8 |  |  | AMOTL2 exon6;NTRK1 exon12 |
|  |  | IRF2BP2 exon1;NTRK1 exon8 |  |  | PRDX1 exon5;NTRK1 exon12 |
|  |  | LRRC71 exon1;NTRK1 exon8 | ④ | **NTRK1 Fusion** | MPRIP exon21;NTRK1 exon14 |
|  |  | LMNA exon2;NTRK1 exon11 |  |  | LMNA exon2;NTRK1 exon16 |
|  |  | LMNA exon3;NTRK1 exon11 | ⑤ | **NTRK2 Fusion** | VCL exon16;NTRK2 exon12 |
|  |  | LMNA exon5;NTRK1 exon11 |  |  | AFAP1 exon13;NTRK2 exon12 |
|  |  | LMNA exon10;NTRK1 exon11 |  |  | VCAN exon6; NTRK2 exon12 |
|  |  | LMNA exon11 del150;NTRK1 exon11 |  |  | NCAA2 exon5;NTRK2 exon13 |
|  |  | PPL exon21;NTRK1 exon11 |  |  | NOS1AP exon9; NTRK2 exon13 |
|  |  | GRIPAP1 exon22;NTRK1 exon11 |  |  | TBC1D2 exon6; NTRK2 exon14 |
|  |  | BCAN exon13;NTRK1 exon11 | ⑥ | **NTRK2 Fusion** | TRIM24 exon12;NTRK2 exon15 |
| ② | **NTRK1 Fusion** | TFG exon5;NTRK1 exon9 |  |  | TRAF2 exon9;NTRK2 exon15 |
|  |  | TPR exon21;NTRK1 exon9 |  |  | SQSTM1 exon4;NTRK2 exon15 |
|  |  | TFG exon4;NTRK1 exon9 |  |  | ETV6 exon5;NTRK2 exon15 |
|  |  | TPM3 exon10;NTRK1 exon9 |  |  | TLE4 exon7;NTRK2 exon15 |
|  |  | AFAP1 exon4;NTRK1 exon9 |  |  | TRIM24 exon12;NTRK2 exon16 |
|  |  | TRIM63 exon8;NTRK1 exon9 |  |  | AGBL4 exon6;NTRK2 exon16 |
|  |  | TPM3 exon8;NTRK1 exon10 |  |  | SQSTM1 exon5;NTRK2 exon16 |
|  |  | SQSTM1 exon2;NTRK1 exon10 |  |  | STRN3 exon7;NTRK2 exon16 |
|  |  | SQSTM1 exon5;NTRK1 exon10 |  |  | WNK2 exon24;NTRK2 exon16 |
|  |  | TPR exon10;NTRK1 exon10 |  |  | QKI exon6;NTRK2 exon16 |
|  |  | TPR exon16 del54;NTRK1 ins13 exon10 |  |  | STRN exon3;NTRK2 exon16 |
|  |  | TPR exon21;NTRK1 exon10 |  |  | GKAP1 exon9; NTRK2 exon16 |
|  |  | CD74 exon8;NTRK1 exon10 |  |  | KCTD8 exon1;NTRK2 exon16 |
|  |  | IRF2BP2 exon1;NTRK1 exon10 |  |  | PRKAR2A exon2;NTRK2 exon16 |
|  |  | IRF2BP2 exon1 del48;NTRK1 exon10 |  |  | PAN3 exon1;NTRK2 exon17 |
|  |  | PPL exon21;NTRK1 exon10 |  |  | SQSTM1 exon5;NTRK2 exon17 |
|  |  | PEAR1 exon15;NTRK1 exon10 |  |  | BCR exon1;NTRK2 exon17 |
|  |  | TFG exon5;NTRK1 exon10 | ⑦ | **NTRK3 Fusion** | ETV6 exon4;NTRK3 exon14 |
|  |  | GRIPAP1 exon22;NTRK1 exon10 |  |  | ETV6 exon5;NTRK3 exon14 |
|  |  | TFG exon6;NTRK1 exon10 |  |  | EML4 exon2;NTRK3 exon14 |
|  |  | F11R exon4;NTRK1 exon10 |  |  | SQSTM1 exon5;NTRK3 exon14 |
|  |  | F11 exon4;NTRK1 exon10 |  |  | TFG exon6;NTRK3 exon14 |
|  |  | SQSTM1 exon6;NTRK1 exon10 |  |  | MYH9 exon31;NTRK3 exon14 |
|  |  | ARHGEF2 exon21;NTRK1 exon10 |  |  | RBPMS exon5;NTRK3 exon14 |
|  |  | CHTOP exon5;NTRK1 exon10 |  |  | BTBD1 exon4; NTRK3 exon14 |
|  |  | NFASC exon21;NTRK1 exon10 |  |  | SPECC1L exon5;NTRK3 exon14 |
|  |  | TPM3 exon7 del39;NTRK1 exon10 |  |  | VIM exon8;NTRK3 exon14 |
|  |  | BCAN exon12;NTRK1 exon10 |  |  | STRN exon3;NTRK3 exon14 |
|  |  | PPL exon11;NTRK1 exon13 |  |  | STRN3 exon3;NTRK3 exon14 |
| ③ | **NTRK1 Fusion** | TPM3 exon8;NTRK1 exon12 |  |  | HNRNPA2B1 exon7;NTRK3 exon14 |
|  |  | LMNA exon6 del172;NTRK1 exon12 |  |  | AKAP13 exon3;NTRK3 exon14 |
|  |  | MPRIP exon21;NTRK1 exon12 |  |  | ETV6 exon5;NTRK3 exon15 |
|  |  | SSBP2 exon12;NTRK1 exon12 |  |  | ETV6 exon4;NTRK3 exon15 |
|  |  | LMNA exon2;NTRK1 exon12 |  |  | SQSTM1 exon6;NTRK3 exon15 |
|  |  | LMNA exon4;NTRK1 exon12 |  |  | ETV6 exon6;NTRK3 exon15 |
|  |  | LMNA exon8;NTRK1 exon12 | ⑧ | **NTRK3 Fusion** | ETV6 exon4;NTRK3 exon12 |
|  |  | LMNA exon10;NTRK1 exon12 |  |  | ETV6 exon5;NTRK3 exon13 |
|  |  | LMNA exon12;NTRK1 exon12 |  |  | ETV6 exon4;NTRK3 exon13 |
|  |  | MPRIP exon14;NTRK1 exon12 |  |  | ETV6 exon5;NTRK3 exon16 |
|  |  | MPRIP exon18;NTRK1 exon12 |  |  |  |

|  |
| --- |
